# Examining changes in parent‐reported child and adolescent mental health throughout the UK's first COVID‐19 national lockdown

**DOI:** 10.1111/jcpp.13490

**Published:** 2021-07-29

**Authors:** Jasmine A. L. Raw, Polly Waite, Samantha Pearcey, Adrienne Shum, Praveetha Patalay, Cathy Creswell

**Affiliations:** ^1^ Department of Psychiatry University of Oxford Oxford UK; ^2^ School of Psychology and Clinical Language Sciences University of Reading Reading UK; ^3^ Department of Experimental Psychology University of Oxford Oxford UK; ^4^ Centre for Longitudinal Studies and MRC Unit for Lifelong Health and Ageing University College London London UK

**Keywords:** COVID‐19, United Kingdom, mental health, children, adolescent

## Abstract

**Background:**

The COVID‐19 pandemic has significantly changed the lives of children and adolescents, forcing them into periods of prolonged social isolation and time away from school. Understanding the psychological consequences of the UK’s lockdown for children and adolescents, the associated risk factors, and how trajectories may vary for children and adolescents in different circumstances is essential so that the most vulnerable children and adolescents can be identified, and appropriate support can be implemented.

**Methods:**

Participants were a convenience sample of parents and carers (*n* = 2,988) in the UK with children and adolescents aged between 4 and 16 years who completed an online survey about their child’s mental health. Growth curve analysis was used to examine the changes in conduct problems, hyperactivity/inattention, and emotional symptoms between the end of March/beginning of April and July using data from monthly assessments over four months. Additionally, growth mixture modelling identified mental health trajectories for conduct problems, hyperactivity/inattention, and emotional symptoms separately, and subsequent regression models were used to estimate predictors of mental health trajectory membership.

**Results:**

Overall levels of hyperactivity and conduct problems increased over time, whereas emotional symptoms remained relatively stable, though declined somewhat between June and July. Change over time varied according to child age, the presence of siblings, and with Special Educational Needs (SEN)/Neurodevelopmental Disorders (ND). Subsequent growth mixture modelling identified three, four, and five trajectories for hyperactivity/inattention, conduct problems, and emotional symptoms, respectively. Though many children maintained ‘stable low’ symptoms, others experienced elevated symptoms by July. These children were more likely to have a parent/carer with higher levels of psychological distress, to have SEN/ND, or to be younger in age.

**Conclusions:**

The findings support previous literature and highlight that certain risk factors were associated with poorer mental health trajectories for children and adolescents during the pandemic.

## Introduction

The COVID‐19 pandemic has caused substantial disruption to children and adolescents through potential threat of illness to themselves and others, school closures, exam disruption, restrictions to social interactions, and increased family pressures (Office for National Statistics, 2020). Pandemic‐associated restrictions have meant that children and adolescents have often experienced prolonged periods of social isolation in addition to increased feelings of uncertainty and high levels of stress amongst the adults around them (Dalton, Rapa, & Stein, 2020).

The most robust evidence to date for the prevalence of mental health difficulties during the pandemic comes from the NHS Digital survey of children and adolescents’ mental health in England (NHS Digital, 2020) that reported that the proportion of children and adolescents with a probable mental health disorder was 1 in 6 in July 2020 (after the end of the national lockdown but while many restrictions were still in place) compared to 1 in 9 in 2017. This deterioration may have been a continuation of the pattern of increasing mental health problems seen in previous surveys; although the finding that over 40% of adolescents reported that the pandemic had made their mental health worse highlights the potential contribution of the pandemic. However, notably, 27.2% of adolescents reported that their mental health had improved during lockdown (Newlove‐Delgado et al., 2021), and self‐report data from over 11,000 pupils aged between 6 and 18 showed that average well‐being remained relatively stable between May and July 2020 (ImpactEd, 2021). Findings across other studies also indicate a mixed picture; while there is some evidence of UK adolescents reporting higher levels of worry and a decline in their mental wellbeing during lockdown (Children’s Parliament, 2020), others suggested that some adolescents ‘thrived’ (Selwyn, 2020) and experienced improvements in their mental health during the first national lockdown (Widnall, Winstone, Mars, Haworth, & Kidger, 2020). For example, around 2,000 young people aged 8–17 years surveyed in June 2020 reported being less frequently stressed over the past month than a previous panel of around 1,850 young people from similar backgrounds, surveyed in March 2020 as the pandemic unfolded (Children’s Commissioner, 2020). Notably, most studies to date have either involved retrospective reports or have compared children’s adjustment between a pre‐lockdown assessment and a single follow‐up assessment during lockdown. There is a lack of empirical and longitudinal research directly examining how mental health symptoms have changed *throughout* the pandemic (Racine et al., 2020) and what might account for differences in children and adolescent’s responses.

There has been particular concern about the mental health impact of the pandemic and associated lockdown restrictions on children who were already vulnerable prior to the pandemic, for example, children and adolescents in low income households (Gutman, Joshi, Parsonage, & Schoon, 2015), with pre‐existing mental health problems (Jefsen, Rohde, Nørremark, & Østergaard, 2020), with SEN (Asbury, Fox, Deniz, Code, & Toseeb, 2021; ImpactEd, 2020) and/or ND (Nonweiler, Rattray, Baulcomb, Happé, & Absoud, 2020), with pre‐existing chronic health conditions (Butler et al., 2018) and where parents experienced high levels of distress (Lawrence, Murayama, & Creswell, 2019). Particular contextual factors may also have created strain on families during the lockdown restrictions. Most notably, being in a single adult or a single child household (Rosen et al., 2020).

In addition to understanding the contextual factors that may increase the risk of a decline in children and adolescents’ mental health throughout the pandemic, it is also critical to explore potentially modifiable factors that have previously been associated with resilience (Fritz, de Graaff, Caisley, van Harmelen, & Wilkinson, 2018). These include individual factors (e.g., cognitive factors and emotion regulation), community factors (e.g., social support), and family factors (Fritz et al., 2018). For example, having a good family climate is associated with a lower prevalence of mental health problems in adolescents (Klasen et al., 2015). Immediate family support has also been shown to weaken the relationship between childhood adversity and the development of emotional symptoms (e.g., depression; Shahar & Henrich, 2016). Given the particular circumstances of the pandemic, when lockdown typically restricted children and young people to being in their family home, we focus here on family factors.

### Present study

In the present study, we aimed to explore the trajectories of change in children and adolescents’ mental health (as reported by their parents/carers) during the UK’s national lockdown in response to the COVID‐19 pandemic. From 23 March until the end June/beginning of July, schools, workplaces, and all non‐essential shops were forced to close, and the public were encouraged to stay at home.

Specifically, we explored the following questions:

1. How did children and young people’s mental health change through the first 4 months of the pandemic?
2. Is change in children and young people’s mental health over time predicted by family contextual and resilience factors?
3. Can we identify children and young people with different sub‐types of trajectories of change in mental health symptoms through this stage of the pandemic?
4. Do family contextual and resilience factors predict the probability of children and young people having these different trajectories?

## Method

### Design

The ‘COVID‐19: Supporting Parents, Adolescents and Children during Epidemics’ (Co‐SPACE) study is an online longitudinal survey composed of a convenience sample of UK parents and carers of children and adolescents aged between 4 and 16 years. The research protocols for the overall Co‐SPACE study and supporting material for this specific project are available via the Open Science Framework (https://osf.io/8zx2y/; https://osf.io/c2v4d/).

### Eligibility

Parents and carers of children and adolescents aged between 4 and 16 years who lived in the UK were eligible to take part.

### Procedure

Participants were invited to report on their child in an online Qualtrics (www.qualtrics.com/uk) survey from 30 March 2020. Parents of multi‐child families were asked to identify one ‘index’ child who they would report on each time. Following completion of the baseline survey, participants were invited back monthly for a follow‐up survey. Informed consent was obtained from the parents/carers. Ethical approval for the study was provided by the University of Oxford Medical Sciences Division Ethics Committee (reference R69060).

### Participants

Participants were eligible parents and carers (aged over 18). The current paper focuses on a sub‐sample of 3,046 out of a total of 5,191^1.^ participants who completed their baseline survey between 30 March and 29 April 2020 and then at least one follow‐up survey between the following dates: 30 April and 31 May (*n* = 2,584); June 1 and June 30 (*n* = 1,825); and July 1 and July 31 (*n* = 1,671).^2.^ Only those who completed the Strengths and Difficulties Questionnaire (SDQ; Goodman, 1997, 2001) and provided full data for the predictor variables (collected at baseline) were included in the analysis (April *n* = 2,988; May *n* = 2,533; June *n* = 1,792 and July *n* = 1,645). Demographic and other baseline information for participants and their children can be found in Table 1. Notably, respondents were predominantly female parents, with higher levels of education, of White ethnicities, and from relatively affluent backgrounds, and while we recruited parents of children aged 4–16 years, the mean age of children was within the primary school age range (around 9 years).

**Table 1:** Demographic and baseline information for participants included in the analyses

	April	May	June	July
	2,988	2,533	1,792	1,645
Parent/carer gender				
Male	170	139	105	110
Female	2,818	2,394	1,687	1,535
Parent/carer ethnicity				
White British	2,880	2,448	1,728	1,589
Other	108	85	64	56
Parent/carer education				
School/vocational qualification	435	373	242	203
Undergraduate degree	1,199	1,007	722	652
Post‐graduate degree	1,335	1,137	822	783
No qualifications	19	16	6	7
Child mean age (*SD*)	9.18 (3.42)	9.15 (3.44)	9.25 (3.40)	9.12 (3.37)
Child gender				
Male	1,557	1,319	932	861
Female	1,431	1,214	861	784
Child ethnicity				
White British	2,776	2,362	1,662	1,533
Other	212	171	130	112
Household income (p.a.)				
<£16,000	148	130	90	71
£16–2,900	318	283	182	163
£30–59,000	888	747	509	478
£60–89,000	682	574	419	390
£90–119,000	375	312	230	209
>£120,000	371	308	238	210
Prefer not to say	206	179	124	124
Family composition				
Single adult household	385	340	229	231
Multiple adult household	2,603	2,193	1,563	1,451
Child Chronic Health				
No chronic health	2,648	2,248	1,581	1,449
Chronic health condition	340	285	211	196
Presence of siblings				
No siblings	737	626	476	435
Siblings	2,251	1,907	1,316	1,210
Mean Depression Anxiety and Stress Scale (DASS; 9 items) (*SD*)	5.20 (4.47)	–	–	–
Mean family warmth (*SD*)	2.71 (0.54)	–	–	–
Mean family conflict (*SD*)	0.86 (0.65)	–	–	–

SEND/ND, Special educational needs/neurodevelopmental disorders.

### Measures

Details and the coding of each measure can be found in the Supporting Information (see Appendix S1).

#### Child and adolescent mental health

The parent‐report version of the SDQ was administered. In the current paper, we focus on the three SDQ sub‐scales that measure mental health symptoms: emotional symptoms, conduct problems, and hyperactivity/inattention.

#### Parent/carer demographic information (baseline survey only)

Parents/carers were asked to report on their own and their child’s age, gender, and ethnicity (see Table 1). We also obtained measures of household income, single adult and single child status, child chronic health, and child SEN and/or ND.

#### Symptoms of psychological distress in parents and carers

A self‐report measure comprising a subset of nine items (McElroy et al., 2020) from the Depression Anxiety Stress Scales (DASS‐21; Lovibond & Lovibond, 1995) was administered.

#### Family support

Family warmth and family conflict were assessed.

### Analysis

Data were organized using R (R Core Team, 2018; v.3.6.2), and analyses were conducted in MPlus (v.8.4; Muthén & Muthén, 2000) and R. Prior to examining the research questions, associations between predictors and baseline SDQ scores were examined to test for potential differences between children at baseline. The first two questions were addressed by specifying latent growth curve models to investigate the change in SDQ scores over time. Linear and non‐linear growth was tested, and time was coded as: 0 (April), 1 (May), 2 (June), and 3 (July). The three SDQ sub‐scales were modelled separately as the dependent variables. In each model, the *intercept* (representing the baseline data) and the *slope* (representing the change over time) of the dependent variables were modelled. Missing data were addressed using full information maximum likelihood estimation (FIML), which uses all information available from all respondents, thus being less prone to biases than a complete case analysis with listwise deletion where the loss of information is larger and would lead to greater biases in estimates. To determine a good statistical fit, we accepted models that had Comparative Fit Measure values >.90 (Kline, 2016) and Root Mean Square Error of Approximation <.08 (Browne & Cudeck, 1993). These models analysed whether the change in the SDQ sub‐scales was predicted by baseline measures of parent/carer psychological distress, family warmth and conflict, child age, gender, ethnicity, chronic health, and SEN/ND as well as total household income (per annum), presence of siblings, and single adult household status.

The third question was assessed using latent growth mixture modelling to identify child‐specific trajectories on outcome measures (the three SDQ sub‐scales). We ran models with an increasing number of trajectories until non‐convergence was reached. Due to negative residual variances and correlations greater than 1 between the latent variables, the slope was constrained to 0 in all models. Model fit was evaluated using Bayesian Information Criterion, the Akaike Information Criteria, entropy index, and the Lo, Mendell, and Rubin (LMR; 2001) statistic (see Table S1). To address the fourth question, class membership was regressed on to the covariates using a multinomial logistic regression (*mlogit* package in R; Croissant, 2020) for each SDQ sub‐scale separately. As all entropy values were <0.80, class probability weights were included in the regression models to account for the lower neatness of classification. The obtained trajectories were compared to the reference group (defined as the largest group) that were expected to be the low symptom groups over time. However, in the multinomial logistic regressions, <£16 k was used as the reference group. Results using the most frequent category (£30–59 k) as the reference group are reported in Appendix S2.

## Results

*Question* *1. How did children and adolescents’ mental health change through the first 4 months of the pandemic?*

*Question* *2. Is change in children and adolescents’ mental health over time predicted by family contextual and resilience factors?*

**Figure 1:**
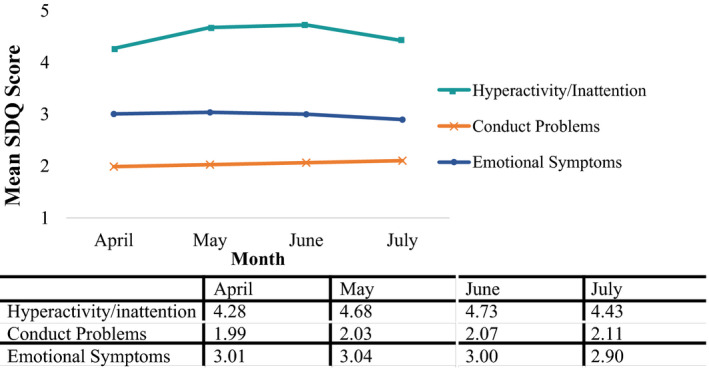
Change in estimated means for the three SDQ sub‐scales between April and July

The latent growth curve analyses explored the changes in children and adolescents’ mental health over the first four months of the pandemic (see Figure 1). Estimating quadratic growth, compared to linear growth, significantly improved the fit for hyperactivity/inattention and emotional symptoms but not for conduct problems. Therefore, we included linear and quadratic growth for hyperactivity/inattention and emotional symptoms but included only a linear growth for conduct problems (see Table 2 for model fit indices).

**Table 2:** Predictors of baseline and change over time in children and adolescents’ for Conduct Problems, Hyperactivity/inattention, and Emotional Symptoms

	Conduct problems		Hyperactivity/inattention		Emotional symptoms	
	*b* (*SE*)	95% CI	*b* (*SE*)	95% CI	*b* (*SE*)	95% CI
Intercept						
DASS	.14 (0.02)***	0.1, 0.17	.16 (0.02)***	0.12, 0.19	.26 (0.02)***	0.22, 0.30
Child ethnicity	.002 (0.02)	−0.03, 0.03	−.01 (0.02)	−0.04, 0.03	−.03 (0.02)	−0.07, 0.003
Child gender	−.06 (0.02)***	−0.09, −0.03	−.17 (0.02)***	−0.2, −0.13	.13 (0.02)***	0.09, 0.16
SEN/ND	.3 (0.02)***	0.26, 0.34	.42 (0.02)***	0.38, 0.46	.3 (0.02)***	0.26, 0.35
Single adult household	−.003 (0.02)	−0.04, 0.03	.01 (0.02)	−0.03, 0.05	.01 (0.02)	−0.04, 0.05
Presence of siblings	.1 (0.02)***	0.07, 0.13	−.02 (0.02)	−0.05, 0.01	.05 (0.02)*	0.02, 0.09
Family warmth	−.2 (0.02)***	−0.24, −0.17	−.1 (0.02)***	−0.14, −0.07	−.03 (0.02)	−0.07, 0.01
Family conflict	.45 (0.02)***	0.42, 0.49	.22 (0.02)***	0.19, 0.26	.12 (0.02)***	0.08, 0.16
Child’s age	−.19 (0.02)***	−0.22, −0.16	−.23 (0.02)***	−0.26, −0.19	−.01 (0.02)	−0.04, 0.03
Child's chronic health	.03 (0.02)	−0.01, 0.06	.03 (0.02)	−0.002, 0.07	.09 (0.02)***	0.05, 0.12
Income: <£16k^a^	.07 (0.02)***	0.03, 0.11	.05 (0.02)*	0.01, 0.08	.06 (0.02)*	0.01, 0.10
Income: £16−29k^a^	.05 (0.02)*	0.01, 0.08	.01 (0.02)	−0.03, 0.05	.03 (0.02)	−0.01, 0.07
Income: £60 – 89^a^	−.03 (0.02)	−0.06, 0.01	−.02 (0.02)	−0.06, 0.02	−.03 (0.02)	−0.07, 0.01
Income: £90−£119^a^	−.01 (0.02)	−0.05, 0.02	−.02 (0.02)	−0.06, 0.02	−.08 (0.02)***	−0.11, −0.04
Income: > £120^a^	−.04 (0.02)*	−0.07, −0.01	−.05 (0.02)*	−0.09, 0.02	−.1 (0.02)***	−0.14, −0.07
Slope						
DASS	.07 (0.06)	−0.05, 0.18	.15 (0.08)	−0.01, 0.30	.08 (0.04)	−0.001, 0.16
Child ethnicity	−.05 (0.05)	−0.15, 0.05	−.01 (0.06)	−0.13, 0.12	−.02 (0.04)	−0.09, 0.05
Child gender	.01 (0.05)	−0.08, 0.11	.001 (0.06)	−0.12, 0.12	−.03 (0.04)	−0.10, 0.05
SEN/ND	−.2 (0.06)*	−0.32, −0.07	−.38 (0.14)*	−0.66, −0.11	−.13 (0.05)*	−0.22, −0.05
Single adult household	.03 (0.06)	−0.09, 0.15	.06 (0.08)	−0.09, 0.21	−.07 (0.05)	−0.16, 0.02
Presence of siblings	−.15 (0.05)*	−0.25, −0.04	−.07 (0.06)	−0.19, 0.05	−.16 (0.04)***	−0.24, −0.07
Family warmth	.09 (0.06)	−0.02, 0.19	−.11 (0.07)	−0.24, 0.03	.003 (0.04)	−0.08, 0.08
Family conflict	−.14 (0.06)*	−0.24, −0.03	.05 (0.06)	−0.07, 0.18	.01 (0.04)	−0.07, 0.10
Child's age	−.05 (0.05)	−0.15, 0.05	−.31 (0.12)*	−0.54, −0.08	−.13 (0.04)*	−0.21, −0.05
Child's chronic health	−.02 (0.05)	−0.12, 0.07	.02 (0.06)	−0.09, 0.14	.04 (0.04)	−0.04, 0.11
Income: <£16 k^a^	−.15 (0.06)*	−0.27, −0.03	−.06 (0.07)	−0.2, 0.09	−.02 (0.05)	−0.11, 0.07
Income: £16–29 k^a^	−.11 (0.06)	−0.23, 0.01	.02 (0.07)	−0.12, 0.15	.01 (0.04)	−0.08, 0.09
Income: £60–89^a^	−.01 (0.06)	−0.12, 0.10	−.1 (0.08)	−0.24, 0.05	−.02 (0.04)	−0.1, 0.07
Income: £90–£119^a^	−.01 (0.05)	−0.11, 0.09	−.09 (0.07)	−0.23, 0.05	−.03 (0.04)	−0.1, 0.05
Income: >£120^a^	.01 (0.05)	−0.1, 0.11	−.07 (0.07)	−0.21, 0.07	.06 (0.04)	−0.02, 0.14
Quadratic						
DASS	–	–	−.09 (0.06)	−0.2, 0.02	−.06 (0.05)	−0.16, 0.03
Child ethnicity	–	–	.004 (0.06)	−0.11, 0.12	.04 (0.04)	−0.04, 0.12
Child gender	–	–	.02 (0.05)	−0.08, 0.12	.02 (0.04)	−0.07, 0.11
SEN/ND	–	–	.3 (0.08)***	0.15, 0.45	.12 (0.05)*	0.02, 0.23
Single adult household	–	–	−.07 (0.07)	−0.2, 0.06	.09 (0.05)	−0.02, 0.19
Presence of siblings	–	–	.05 (0.05)	−0.06, 0.15	.15 (0.05)*	0.06, 0.24
Family warmth	–	–	.08 (0.06)	−0.04, 0.19	.03 (0.05)	−0.07, 0.12
Family conflict	–	–	−.08 (0.06)	−0.19, 0.03	−.03 (0.05)	−0.13, 0.06
Child’s age	–	–	.29 (0.07)***	0.14, 0.43	.15 (0.05)*	0.06, 0.25
Child’s chronic health	–	–	.001 (0.05)	−0.10, 0.10	−.02 (0.05)	−0.11, 0.07
Income: <£16 k^a^	–	–	.05 (0.06)	−0.08, 0.17	.03 (0.06)	−0.08, 0.13
Income: £16–29 k^a^	–	–	−.02 (0.06)	−0.14, 0.10	−.02 (0.05)	−0.11, 0.08
Income: £60–89^a^	–	–	.08 (0.06)	−0.04, 0.20	.04 (0.05)	−0.06, 0.14
Income: £90–£119^a^	–	–	.08 (0.06)	−0.04, 0.20	.05 (0.05)	−0.04, 0.14
Income: >£120¹	–	–	.07 (0.06)	−0.05, 0.18	−.05 (0.05)	−0.14, 0.05
Comparative Fit Index (CFI)		0.983	–	0.998	–	0.999
Robust Tucker–Lewis Index (TLI)		0.967	–	0.992	–	0.996
Root Mean Square Error of Approximation (RMSEA)		0.035	–	0.018	–	0.012

*p < .05; **p < .01; ***p < .001.

^a^ Income reference group = £30–59 k.

When examining the overall change in SDQ scores for the whole group over time, levels of hyperactivity/inattention and conduct problems increased across the whole group over time (see Figure 1). Levels of hyperactivity/inattention particularly increased between April and May before decreasing between June and July. Conversely, emotional symptoms remained relatively stable over time, though declined somewhat between June and July.

Across all three models, higher baseline levels of parent/carer psychological distress, higher levels of family conflict between the parent/carer and their child, having SEN/ND, having a sibling (with the exception of hyperactivity/inattention), and having an annual income of <£16 k were all associated with higher baseline levels of emotional symptoms, conduct problems, and hyperactivity/inattention (see Table 2). Conversely, warmer parent/child relationships were associated with lower baseline levels of conduct problems and hyperactivity/inattention. Meanwhile, female compared to male children had lower baseline levels of conduct problems and hyperactivity/inattention but higher emotional symptoms. Children with a chronic health condition, compared to those without, also had higher baseline levels of emotional symptoms. Finally, children from families with higher incomes were reported to have lower baseline levels of conduct problems (annual income >£120 k), emotional symptoms (annual income >£90 k), and hyperactivity/inattention (annual income >£120 k).

### Changes in conduct problems

Many of the variables that were associated with elevated scores at baseline were associated with a slower rate of increase in symptoms over the lockdown period (see Table 2). This was the case for children with SEN/ND, those who had siblings, those with higher levels of family conflict, and those from families earning < £16k.

### Changes in hyperactivity/inattention

Children without SEN/ND (who had lower baseline hyperactivity/inattention compared to those with SEN/ND) exhibited a quadratic growth, whereby they initially experienced an increase between April and May followed by a decrease between June and July (see Table 2; Figure S1A). Likewise, younger children, who initially had higher initial levels compared to older children, showed a similar quadratic pattern between April and May and between June and July (see Figure S1B).

### Changes in emotional symptoms

Children with SEN/ND (who had higher baseline emotional symptoms compared to children without SEN/ND) experienced an overall decrease in emotional symptoms over lockdown with a particularly steep decrease between April and May (see Table 2; Figure S2A). The opposite pattern was seen for younger children and those without siblings (see Figure S2B,C). Younger children, who initially had higher levels of emotional symptoms than older children, showed an increase between April and May followed by a decrease between June and July. Similarly, children without siblings (who had lower baseline emotional symptoms compared to children with siblings) experienced an increase between April and May and a decrease between June and July.

*Question* *3. Can we identify children and adolescents with different sub‐types of trajectories of change in mental health symptoms through this stage of the pandemic?*

*Question* *4. Do family contextual and resilience factors predict the probability of children and young people having these different trajectories?*

#### Group Trajectories of conduct problems

**Figure 2:**
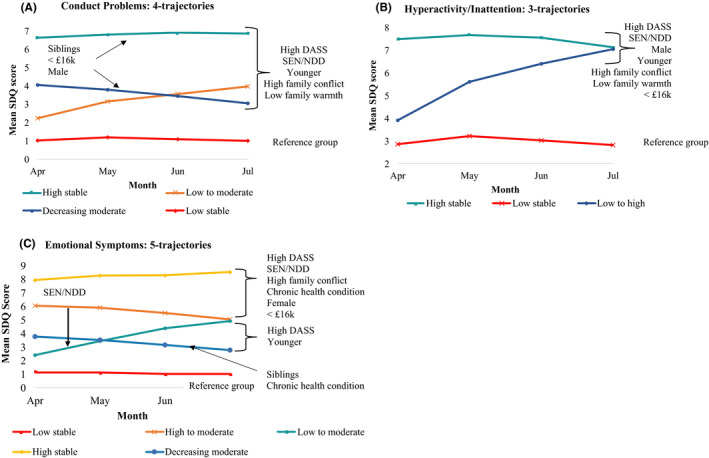
Symptom trajectories for (A) Conduct Problems, (B) Hyperactivity/Inattention and (C) Emotional Symptoms in children and adolescents throughout the first four months of the UK’s national lockdown

A four‐trajectory model was chosen for further exploration (see Table 3; see Table S1 for model fit criteria) that identified a low stable (the reference group; *n* = 2,042), a low to moderate (*n* = 407), a decreasing moderate (*n* = 416), and a high stable symptom group (*n* = 123; see Figure 2A). Compared to the reference group, children and adolescents within all the groups that did not have low scores by July (i.e., the high stable, low to moderate, and decreasing moderate groups) were more likely to have parents with higher levels of baseline psychological distress, to have SEN/ND, or to come from families with higher levels of reported family conflict (stable high group: Odds Ratios OR [95% CI] = 1.12 [1.08–1.17], 15.17 [9.41–24.46], and 11.34 [8.24–15.61], respectively; low‐to‐moderate group ORs = 1.07 [1.05–1.10], 2.21 [1.55–3.15] and 2.86 [2.29–3.57], respectively; decreasing moderate group ORs = 1.09 [1.06–1.12], 6.46 [4.73–8.83], and 5.88 [4.68–7.40], respectively), but were less likely to be older or to come from families with higher levels of family warmth (stable high group ORs = 0.87 [0.81–0.92] and 0.25 [0.18–0.34], respectively; decreasing moderate group ORs = 0.86 [0.83–0.90] and 0.43 [0.35–0.54], respectively; low‐to‐moderate group ORs = 0.85 [0.82–0.89] and 0.53 [0.42–0.66], respectively).

**Table 3:** Sample breakdown and intercept and slope coefficients by trajectory group

	Trajectory	*N* (%)	Intercept	Slope (*p*)
Conduct problems	1 High Stable	123 (4.11)	6.67	0.10
	2 Low to Moderate	407 (13.62)	2.37	0.59***
	3 Decreasing Moderate	416 (13.92)	4.10	−0.34***
	4 Low Stable^a^	2,042 (68.34)	1.10	−0.01
Hyperactivity/inattention	1 High Stable	812 (27.18)	7.65	−0.11**
	2 Low Stable^a^	1,976 (66.13)	3.01	−0.004
	3 Low to High	200 (6.69)	4.17	1.07***
Emotional symptoms	1 Low Stable^a^	1,473 (49.30)	1.16	−0.07***
	2 High to Moderate	440 (14.73)	6.14	−0.33***
	3 Low to Moderate	275 (9.20)	2.51	0.87***
	4 High Stable	202 (6.80)	7.98	0.18**
	5 Decreasing Moderate	598 (20.00)	3.82	−0.33**

^a^ Reference groups.

In addition, children and adolescents within the high stable and decreasing moderate groups were more likely than the reference group to have siblings (ORs = 3.30 [1.85–5.88] and 1.74 [1.27–2.38], respectively) but less likely to be female (stable high group OR = 0.61 [0.39–0.94] and decreasing moderate group OR = 0.69 [0.53–0.89]). Additionally, they were less likely to come from families earning: £30–59 k, £60–89 k, or £120 k+ than from families earning < £16 k (high stable symptom group ORs = 0.35 [0.15–0.79], 0.26 [0.1–0.63], and 0.13 [0.04–0.45], respectively).

#### Trajectories of hyperactivity/inattention

A three‐trajectory model was chosen for further exploration (see Table 3; see Table S1 for model fit criteria) that identified a low stable (the reference group; *n* = 1,976), high stable (*n* = 812), and a low to high group (*n* = 200; see Figure 2B). Compared to the low stable group, children in the high stable and low to high group were more likely to have parents with higher levels of psychological distress, to have SEN/ND, or to come from families with higher levels of reported family conflict (high stable group ORs = 1.11 [1.09–1.14], 11.94 [9.08–15.71], and 2.28 [1.93–2.69], low to high group ORs = 1.07 [1.03–1.11], 2.04 [1.19–3.49], and 1.54 [1.18–2.02]) but less likely to be female, older, or from families with higher levels of family warmth (high stable group ORs = 0.46 [0.37–0.56], 0.83 [0.8–0.85], and 0.63 [0.52–0.77], low to high group ORs = 0.69 [0.5–0.95], 0.89 [0.84–0.94], and 0.67 [0.5–0.91]). In addition, children within the high stable group were less likely to be from families earning £60–89 k, £90–119 k, or £120 k+ than from families earning <£16 k (ORs = 0.55 [0.33–0.91], 0.56 [0.33–0.97], and 0.47 [0.27–0.81], respectively).

#### Trajectories of emotional symptoms

A five‐trajectory model was chosen for further exploration (see Table 3; see Table S1 for model fit criteria) that identified a low stable (the reference group; *n* = 1,473), a low to moderate (*n* = 275), a high to moderate (*n* = 440), decreasing moderate (*n* = 598), and a high stable group (*n* = 202; see Figure 2C). Children in the groups that had high symptom scores at baseline (i.e. within the high stable and high‐to‐moderate symptom groups) were more likely to have parents/carers with higher levels of baseline psychological distress, to have SEN/ND, to come from families with higher levels of reported family conflict, to have a chronic health condition or to be female (high stable ORs = 1.24 [1.2–1.28], 11.94 [8.17–17.44], 1.77 [1.4–2.24], 2.90 [1.92–4.38], and 2.79 [1.99–3.92], respectively, and high‐to‐moderate ORs = 1.19 [1.16–1.22], 4.84 [3.55–6.58], 1.64 [1.37–1.97], 2.07 [1.46–2.93], and 1.76 1.39–2.24], respectively). Children within the high stable group were less likely to come from families earning £30–59 k, £60–89 k, £90–119 k, or £120 k+ than from families earning <£16 k (ORs = 0.41 [0.21–0.8], 0.35 [0.17–0.72], 0.16 [0.07–0.4], and 0.17 [0.07–0.4], respectively). Similarly, children in the high‐to‐moderate group were less likely to come from families earning £60–89 k, £90–119 k, or £120 k+ than from families earning <£16 k (ORs = 0.49 [0.28–0.88], 0.37 [0.19–0.69], and 0.26 [0.13–0.5], respectively).

Children in the groups that had scores within the moderate range at the endpoint (i.e. within the decreasing moderate and low‐to‐moderate groups) compared to the reference group were also more likely to have parents/carers with higher levels of baseline psychological distress (ORs = 1.12 [1.09–1.15] and 1.13 [1.09–1.16], respectively), SEN/ND (decreasing moderate group OR = 2.47 [1.8–3.38]), or a chronic health condition (low‐to‐moderate group OR = 1.81 [1.17–2.8]). They were however, less likely to be older in age (OR = 0.93 [0.9–0.96] and 0.95 [0.91–0.99], respectively) or to have siblings (low‐to‐moderate OR = 0.67 [0.49–0.91]).

## Discussion

This study aimed to understand how children and adolescents’ parent reported mental health changed among a convenience sample who participated in an online survey through the first 4 months of the COVID‐19 pandemic and if the change could be predicted by family contextual and resilience factors. We also sought to identify children and adolescents who may have experienced different patterns of trajectories in mental health symptoms and whether family contextual and resilience factors predicted the likelihood of children and adolescents having these different trajectories.

The overall pattern of change over time for hyperactivity/inattention (and to a lesser extent) emotional symptoms among our sample mirrored the national restrictions, with increases in symptoms when families were most restricted (in April and May) and a reduction as restrictions eased somewhat (in June and July); although, notably, the later reduction was not seen for conduct problems. The marked changes in hyperactivity/inattention symptoms (that were seen particularly among younger boys) are notable given the particular increase in ‘probable’ disorders in this domain seen in the national survey of children and young people’s mental health in England conducted in 2020, compared to previous findings in 2017, and give weight to the likelihood that the national survey findings at least in part reflected pandemic related restrictions, and potentially may not have reflected the peak level of difficulties (given that data was collected in July 2020).

Consistent with the previous research, our findings highlight several characteristics and/or contexts that are consistently associated with higher risk of mental health problems among children and adolescents; specifically, the presence of child SEN/ND (Wang, Zhang, Zhao, Zhang, & Jiang, 2020), elevated parents/carers mental health symptoms (Lawrence et al., 2019), higher levels of family conflict, and low family incomes (Gutman et al., 2015). Notably, many of these variables were associated with a slower rate of increase in conduct problems over time, which suggests that these were not necessarily vulnerability factors in the specific lockdown context. It is also important to note that some children and adolescents experienced an overall reduction in their mental health symptoms over time (e.g. children with SEN/ND). This supports previous findings suggesting that some children and adolescents may have experienced some small benefits of the lockdown measures that were implemented (Asbury et al., 2021); possibly because staying at home provided some children with a less stressful environment, particularly for those who experienced difficulties in school previously. However, it is important to note that, although some groups experienced reductions in symptoms over time, their symptoms remained elevated throughout the study.

Conversely, there were some children and adolescents who showed an overall increase in mental health symptoms over time. Notably, parents/carers reported lower baseline emotional symptoms for only children than for children with siblings but an overall greater increase over time; particularly increasing between April and May (when schools were closed, socializing was restricted to household members only, and outdoor exercise was limited to one session per day). These findings suggest that only children may have been particularly vulnerable to the negative consequences of lockdown measures; potentially because of a heightened risk of loneliness due to not being in school and around peers (Loades et al., 2020). Indeed, the presence of a sibling has previously been shown to be a protective factor for children experiencing stressful life events (Gass, Jenkins, & Dunn, 2007).

It is important to recognize that the general patterns seen across the whole sample may mask different patterns in the trajectories of symptoms over time. Our second set of analyses identified stable (high or low) trajectories for some children and adolescents, but increasing or decreasing trajectories for others (although decreasing trajectories typically remained elevated). It is notable that, across all subscales, many children had ‘stable low’ symptoms (49%–68%); however, children and adolescents with elevated symptoms by the end of the study (regardless of baseline symptoms) were more likely to be younger or to have a parent with higher self‐reported mental health symptoms (of depression, anxiety, and stress) or SEN/ND. For conduct problems and hyperactivity symptoms (but not emotion), they were also more likely to have higher levels of family conflict and lower family warmth. Notably, those with siblings were more likely to be in the high stable group for conduct problems, although given this scale includes an item about fighting with other children, it is possible that this, at least to some extent, reflects the greater opportunity for fighting among those with siblings.

Groups experiencing reductions in symptoms (but still elevated) over time did not differ from groups who had increasing or stable high symptoms in the characteristics we examined. Like those with stable high symptoms, children and adolescents who experienced reductions in all sub‐scales were more likely to have a parent/carer with higher mental health symptoms, to have SEN/ND, or to come from a family with high reported levels of family conflict than the stable low groups. Overall, the associations between family factors (including income, parent mental health, and family relationships) and child outcomes during the pandemic mirror previous findings from non‐pandemic contexts (e.g. Fritz et al., 2018; Gutman et al., 2015) and further emphasize the importance of policy and practice that supports families and enables and empowers parents to be able to support their children – a need that is likely to be especially important in lockdown contexts when children have limited access to support outside the home.

Collectively, our results highlight that mental health trajectories throughout the first 4 months of the pandemic within the UK were varied. Some families may have benefitted from the lockdown measures by spending more time together and building stronger relationships within families (Clayton, Clayton, & Potter, 2020) or avoiding school environment‐related stressors (Hoekstra, 2020). For example, in March 2020, the Children’s Commissioner for England’s report found that 75% of responses to an open question about sources of stress mentioned school‐related stressors, such as being distracted by other students, homework, and studying for exams, whereas 3 months later, only 46% of respondents mentioned stressors related to school (Children’s Commissioner, 2020). However, others may have experienced increased pressures associated with lockdown, such as elevated stress among parents due to the competing demands of work and home‐schooling (Office for National Statistics, 2020). This may have been particularly the case for families with younger children where we saw poorer mental health trajectories than adolescents. This may reflect the particular demands on families with younger children who are likely to be more dependent on parents/carers for maintaining contact with peers, completing home‐schooling, and meeting their day‐to‐day needs. On the other hand, adolescents may have been able to maintain better mental health over time, including by staying connected to peers albeit remotely. Furthermore, parents with younger children may have been influenced by their own feelings of stress while juggling home‐schooling and work responsibilities that may have amplified their negative perception of their children’s behaviours. We prioritized parent report so that we could examine changes in mental health in children and young people across a broad age range; however, our reliance on parent‐reported data introduces the possibility that parents were less reliable reporters of adolescents’ (compared to children’s) mental health, and particularly, emotional (Van Roy, Groholt, Heyerdahl, & Clench‐Aas, 2010) symptoms; possibly incorrectly estimating their severity (van der Meer, Dixon, & Rose, 2008). Going forwards, it will be important to triangulate these findings with others that have prioritized young people’s self‐report.

Although this study has the strength of having monthly measures throughout this unprecedented period, some limitations need to be considered. First, we did not obtain pre‐pandemic data which makes it difficult to know if the severity of mental health symptoms among this population increased or decreased *as a result of* lockdown nor do we know the extent to which our baseline measures had already been affected by experiences of COVID‐19 up to that point. Second, it is important to highlight that our sample was opportunistic and not representative. In other words, we do not know how many people chose not to participate in the study, and the recruitment approach will likely have attracted volunteers who are already well engaged and interested and relied on internet access (e.g. Pierce et al., 2020). Bias in our sample is clear towards families on higher annual incomes and those from White British backgrounds as well as those with younger children. It is also notable that our mean SDQ scores in July are higher than those found in a nationally representative survey of children and adolescents’ mental health that was conducted during the same month (with our mean scores being about 0.6 points higher across measures). Therefore, we cannot generalize our results to the broader experiences of UK families. As such, while the findings are useful in providing information on the sorts of characteristics that are associated with change over time during the pandemic, they should not be used to draw conclusions about population‐level prevalence. Third, like most longitudinal studies, our study suffers from attrition over timepoints, and in our study, this loss to follow‐up is non‐monotonic (i.e., parents might miss a timepoint and then participate in subsequent timepoints). We used principled approaches to dealing with the missing data in analysis such as full‐information maximum likelihood; however, it is important to note that attrition was more likely based on disadvantaged socio‐economic circumstances and poorer mental health, and this might have led to underestimation of some associations that we report in this study. Fourth, our measures of family conflict and family warmth only consider the child and parent/carer relationship and not the wider family dynamics. This is an important limitation given evidence that family support as a whole, rather than from one parent/carer, was a more important resilience factor in moderating the relationship between childhood adversity and psychopathology (Fritz et al., 2018). Finally, we did not consider whether direct or indirect exposure to COVID‐19 influenced the trajectories of children and adolescents’ mental health symptoms. It is likely that experiences of a bereavement, or having a critically ill family member, may have adversely affected children and adolescent’s mental health. Other factors that may well have also influenced these trajectories include access to outdoor spaces, opportunities to engage in activities that improve mental health, and the child’s relationship with school work. Furthermore, while we have data from the four countries within the UK, we acknowledge that the restrictions and infection rates differed across and between them, particularly at later points of the study.

Despite these limitations, our findings emphasize the varied outcomes experienced by different children and adolescents during the pandemic and the importance of understanding family contextual and resilience factors in the mental health trajectories for children and adolescents during a pandemic. As has often been said during the pandemic, ‘we are all in the same storm, but are in very different boats’.

## Supporting information


**Appendix S1.** Additional details of each measure including the coding of variables in the models.
**Appendix S2.** Multinomial logistic regression results using £30–59k as the reference group.
**Table S1.** Model Fit Information for the Growth Mixture Models.
**Figure S1.** Change in estimated means for hyperactivity/inattention.
**Figure S2.** Change in estimated means for emotional symptoms.

## Notes

1 Of the 5191 participants who completed the baseline survey, 2,145 did not return to complete a survey between May and July.

2 For participants who had multiple entries per calendar month, only the first entry was used in the analysis.
