# Supplementary material for: Examining changes in parent‐reported child and adolescent mental health throughout the UK's first COVID‐19 national lockdown
Source: J Child Psychol Psychiatry. 2021 Jul 29;62(12):1391–401. doi: 10.1111/jcpp.13490 (PMC8447308; doi:10.1111/jcpp.13490)
Supplement: Supplementary file 1 — Appendix S1. Additional details of each measure including the coding of variables in the models. Appendix S2. Multinomial logistic regression results using £30–59k as the reference group. Table S1. Model Fit Information for the Growth Mixture Models. Figure S1. Change in estimated means for hyperactivity/inattention. Figure S2. Change in estimated means for emotional symptoms. [file JCPP-62-1391-s001.docx]

**Supporting Information**

**Examining changes in parent-reported child and adolescent mental health throughout the UK’s first COVID-19 national lockdown – by Raw *et al.***

Appendix S1. Additional details of each measure including the coding of variables in the models

Appendix S2. Multinomial logistic regression results using £30-59k as the reference group

Table S1. Model Fit Information for the Growth Mixture Models

Figure S1. Change in estimated means for hyperactivity/inattention

Figure S2. Change in estimated means for emotional symptoms

Supplemental References

**Appendix S1. Additional details of each measure including the coding of variables in the models**

*Child and adolescent mental health.* The parent-report version of the SDQ was used to measure the mental health of children and adolescents. It contains a total of 25 items that are sub-divided into 5 scales reflecting: emotional symptoms, conduct problems, hyperactivity/inattention, peer relationship problems and prosocial behaviour. Each statement is rated using a 3-point Likert scale ranging from 0 (“*not at all*”) to 2 (“*certainly true*”) and parents/carers rate how true each statement is of their child. A score for each sub-scale is obtained by summing the responses in each of the sub-scales when at least 3/5 items are completed. For occasions where there are at least 3/5 items completed but also missing data, scores are scaled up pro rata. Each sub-scale will therefore have a maximum score of 10. The parent version of the SDQ has been shown to have satisfactory psychometric properties (Goodman, 2001; Reardon, Spence, Hesse, Shakir, & Creswell, 2018; Stone, Otten, Engels, Vermulst, & Janssens, 2010)

*Symptoms of psychological distress in parents and carers.*  Parents responded to a sub-set of 9 items from DASS-21 using a 4-point severity/frequency scale ranging from 0 (“*Did not apply to me at all”)* to 3 (“*Applied to me very much”*). An overall score was obtained by summing the scores for each item with a potential total score between 0 and 27.

*Family support.* Measures of family warmth and family conflict were obtained using responses to the following statements: *“My child and I have a warm, close relationship”*, and *“My child and I argue a lot”.* Each statement was rated on a 4-point Likert scale ranging from 0 (*“Not at all”*) to 3 (*“Completely”*).

*Parent/ carer’s total household income*. Parents/carers were asked to indicate their total annual household income by selecting one of the following options: “*Less than £16,000 a year (£310 a week)”, “£16,000-£29,999 a year (£310-£569 a week)”, “£30,000-£59,999 a year (£569-£1149 a week)”, “£60,000-£89,999 a year (£1500-£1729 a week)”, “£90,000-£119,999 a year (£1730-£2299 a week)”, “More than £120,000 a year (£2300 a week)”, “Prefer not to say”.* As there was a sufficient number of responses within each income category to be included in the model as a separate group, 5 dummy variables were created. Each was contrasted against the group with the largest *n: “£30,000-£59,999 a year (£569-£1149 a week)”*. Responses of “*Prefer not to say”* were treated missing and a single imputation procedure was carried out in Mplus using the *“Impute*” function.

*Child’s gender.* Parents and carers were asked to indicate their child’s gender: “*Male*”, “*Female*” or “*Other/ prefer not to say*”. Those who selected “Other/ prefer not to say” were not included in the analysis due to the small sample size. Children’s gender was coded as: Male = 0; Female = 1.

*Child’s ethnicity.* Parents and carers were asked to indicate their own ethnicity from the following options: “*Asian/British – Indian, Pakistani, Bangladeshi*, *other”,* “*Black/Black British – Caribbean, African, other*”, “*Mixed race – White and Black/Black British*”, “*Mixed race – other*”, “*White – British, Irish, other*”, “*Chinese/Chinese British*”, “*Middle Eastern/Middle Eastern British – Arab, Turkish, other*”, “*Other ethnic group*”, or “*Prefer not to say*”. Subsequently, they were asked to indicate if their child shared the same ethnicity as their own and if not, to indicate their child’s ethnicity using the same aforementioned choices. As there were not enough responses in each category to be included in the model separately, a dichotomous variable was created to represent ethnicity: 0 = Non-White – British, Irish, other; 1 = all other ethnic groups combined.

*Health-related factors of child.* Parents and carers were asked “*Does anyone in your household have any of the following medical conditions?*” with the option to answer in relation to whether the index child had a range of physical health, mental health, and neurodevelopmental conditions. In addition, parents and carers were also asked *“Does your child have any special educational needs?”.*

*Child chronic physical health conditions.* If the parent or caregiver selected at least one of the following multiple choice options to the aforementioned question: *high blood pressure”, “diabetes”, “heart disease, “lung disease (e.g. asthma or COPD)”, “cancer”, “another clinically-diagnosed ill physical health condition”, “a disability that affects my ability to leave the house”,* or *“any other disability”* then their child was classed as having a chronic physical health condition. A dichotomous variable was created to represent chronic health: 0 = no chronic illness; 1 = chronic illness.

*SEN/ Neurodevelopmental disorders.* If the parents of caregivers selected at least one of the following multiple choice options to the aforementioned question: *“attention-deficit disorder (ADD)/attention-deficit hyperactivity disorder (ADHD)”,* or *“autism spectrum disorder (ASD)”* then their child was classed as having a neurodevelopmental disorder*.* If they responded “yes” to the question about special educational needs, they were further asked to specify what special education needs their child has from the following options: *“communicating and interacting”*, *“cognition and learning”*, *“social, emotional and mental health difficulties”* or *“sensory and/or physical needs”.* Given the anticipated high degree of overlap, the responses to these two questions were combined to create one variable which represents a child having special educational needs and/or a neurodevelopmental disorder: 0 = no SEN/ND, 1 = SEN/ ND.

*Presence of siblings.* Parents/carers are asked to indicate whether the child had siblings and if so, how many (including step and foster siblings) were living in the household. A dichotomous variable was created to reflect the presence of siblings: 0 = no siblings; 1 = one sibling or more.

*Single adult household.* Parents/carers are asked to list the ages of all other members of their household (in years). A single-adult household was be defined as a household in which the parent/carer taking the survey was the only adult over the age of 18 years. A dichotomous variable to reflect single-adult households was created: 0 = not a single adult household; 1 = single adult household.

**Appendix S2. Multinomial logistic regression results using £30-59k as the reference group**

*Group Trajectories of conduct problem, hyperactivity/inattention and emotional symptoms.* In order to examine the differences between income categories and the likelihood of being in the reference group or another trajectory group for each sub-scale, the multinomial logistic regressions were also run using < £30-59k income category as the reference group.

*Conduct problems.* Children within the high stable group were more likely than the reference group to come from families earning < £16k or £16-£29k than from families earning £30-£59k (ORs = 2.89 and 2.17, respectively). Additionally, children within the low to moderate group were less likely to come from families earning £16-£29k than from families earning £30-£59k (OR = .62).

*Hyperactivity/inattention.* Children within the high stable group were less likely to be from families earning £60-89k or £120k+ than from families earning £30-£59k (ORs = .72 and .61, respectively).

*Emotional symptoms.* Children within the high stable group were more likely to be from families earning the lowest annual income (< £16k; OR = 2.42) but less likely to be from families earning £90-119k or £120k+ than from families earning £30-£59k (ORs = .39 and .40). Similarly, children within the high to moderate group were also less likely to be from families earning £90-119k or £120k+ than from families earning £30-£59k (ORs = .59 and .42). Finally, children in the decreasing moderate group were less likely to come from families earning £120k+ than from families earning £30-£59k (OR =.68).

| **Table S1.** |  |  |  |  |  |  |
| --- | --- | --- | --- | --- | --- | --- |
| *Model Fit Information for the Growth Mixture Models* | | | | | | |
| SDQ Subscale | Number of trajectories |  |  |  |  | LMR LRT *p-*value |
|  |  | Entropy |  | AIC | BIC |  |
| Conduct problems | 2 | 0.85 |  | 29943.73 | 30003.75 | 0.000 |
|  | 3 | 0.78 |  | 29734.90 | 29812.93 | 0.000 |
|  | 4 | 0.75 |  | 29536.03 | 29632.06 | 0.000 |
|  | 5 | 0.77 |  | 29473.79 | 29587.83 | 0.164 |
| Emotion Symptoms | 2 | 0.77 |  | 36027.15 | 36087.17 | 0.000 |
|  | 3 | 0.78 |  | 35831.48 | 35909.51 | 0.000 |
|  | 4 | 0.74 |  | 35578.57 | 35674.60 | 0.000 |
|  | 5 | 0.74 |  | 35499.44 | 35613.48 | 0.032 |
|  | 6 | 0.75 |  | 35433.41 | 35565.46 | 0.326 |
| Hyperactivity/  Inattention | 2 | 0.64 |  | 36723.26 | 36783.28 | 0.000 |
|  | 3 | 0.66 |  | 36599.01 | 36677.04 | 0.000 |
|  | 4 | 0.64 |  | 36530.26 | 36626.30 | 0.154 |

Figure S1. Change in estimated means for hyperactivity/inattention between April and July for A) SEN/ND vs. No-SEN/ND and B) Age


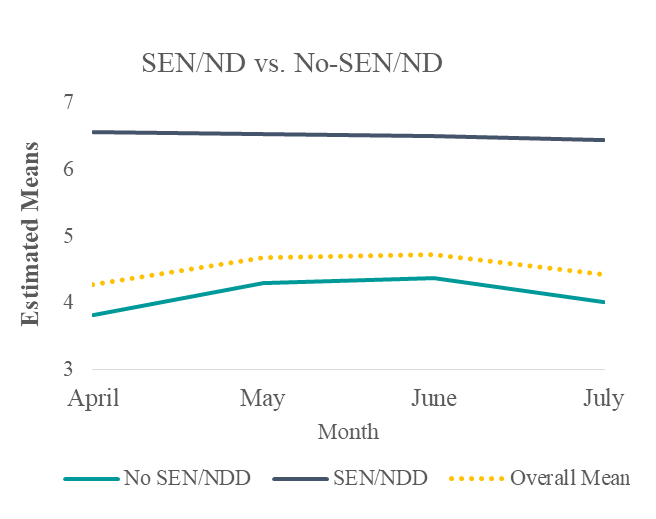


A


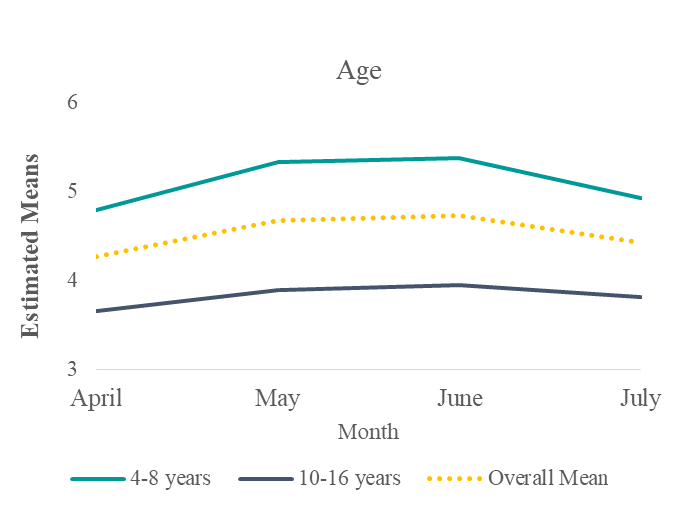


B


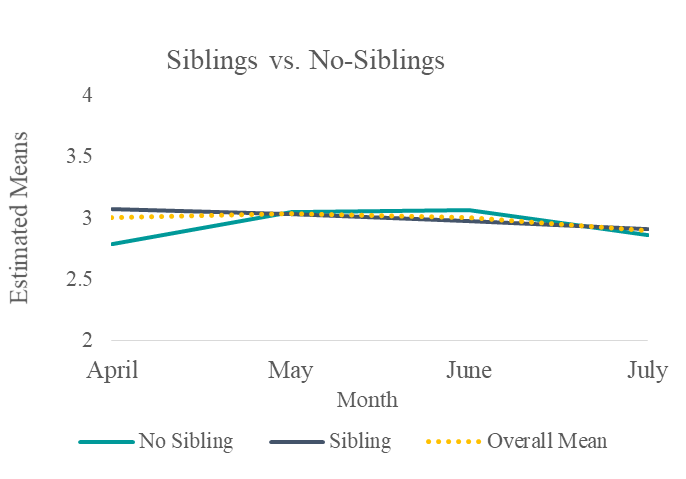

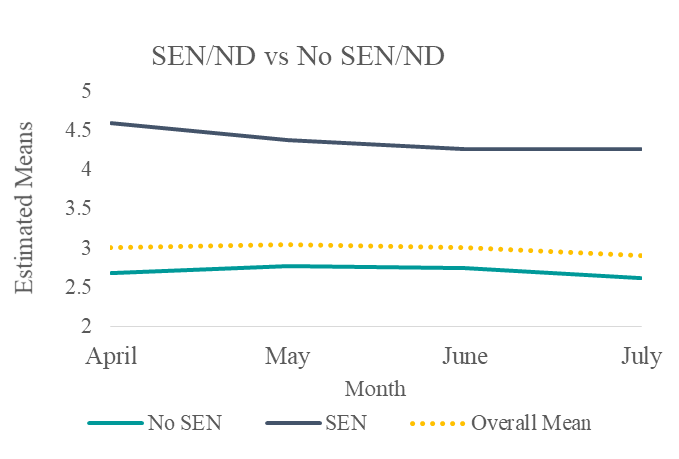


Figure S2. Change in estimated means for emotional symptoms between April and July for A) SEN/ND vs. No-SEN/ND, B) Age and C) Siblings vs. No-Siblings


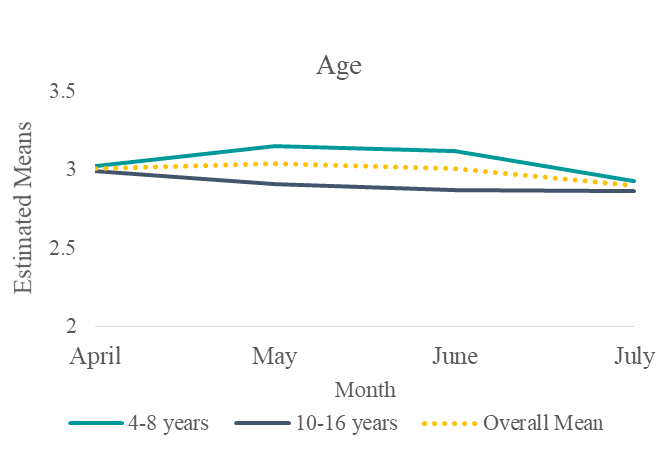


C

B

A

**Supplemental References**

Goodman, R. (2001). Psychometric properties of the strengths and difficulties questionnaire. *Journal of the American Academy of Child and Adolescent Psychiatry*.

Reardon, T., Spence, S. H., Hesse, J., Shakir, A., & Creswell, C. (2018). Identifying children with anxiety disorders using brief versions of the Spence Children’s Anxiety Scale for children, parents, and teachers. *Psychological Assessment*.

Stone, L. L., Otten, R., Engels, R. C. M. E., Vermulst, A. A., & Janssens, J. M. A. M. (2010). Psychometric properties of the parent and teacher versions of the strengths and difficulties questionnaire for 4- to 12-Year-olds: A review. *Clinical Child and Family Psychology Review*.
